# A High-Frame-Rate Display Method for Multiple Synergistic Digital Micromirror Devices Involving Large Target Surfaces

**DOI:** 10.3390/mi17020189

**Published:** 2026-01-30

**Authors:** Zheng Liu, Yingjie Wang, Jie Li, Xiayang Huang, Pengxi Liu, Wennan Cui, Tao Zhang

**Affiliations:** 1 School of Information Science and Technology, ShanghaiTech University, Shanghai 201210, China; liuzheng@shanghaitech.edu.cn (Z.L.); wangyj22023@shanghaitech.edu.cn (Y.W.); 2 Shanghai Institute of Technical Physics, Chinese Academy of Sciences, Shanghai 200083, China; lijie183@mails.ucas.ac.cn (J.L.); huangxiayang@mail.sitp.ac.cn (X.H.); ccliuwei@sina.com (P.L.); cuiwennan@mail.sitp.ac.cn (W.C.); 3 University of Chinese Academy of Sciences, Beijing 100049, China

**Keywords:** digital micromirror device, large-target-surface, high-frame-rate, synchronized display, semi-physical simulation

## Abstract

This study proposed a large-target-surface and high-frame-rate display method using multiple Digital Micromirror Devices (DMDs) for high-resolution, high-frame-rate aerospace applications. DMDs offer high frame rates and contrast ratios, but their surface size is constrained. By employing Pulse Width Modulation (PWM) with synchronization signals for grayscale modulation and synchronizing multiple DMDs, this method achieved a target surface four times larger than a single DMD at 400 Hz frame rate, with synchronization errors below 10 ns. This enhances simulation efficiency and provides an effective infrared scene simulation solution.

## 1. Introduction

With the rapid advancement of aerospace technology, there is an ever-increasing demand for large-format, high-frame-rate detection systems [1,2,3,4]. Such systems encounter significant challenges in functional and performance testing, with the core bottleneck being the difficulty in reproducing representative real-world operating environments under laboratory conditions. In typical application scenarios such as satellite remote sensing and airborne electro-optical reconnaissance pods, the scale of large-format detectors has reached 1920×1080 and beyond, and the targets to be detected usually exhibit high-speed dynamic characteristics [5]. When a target undergoes a relatively large angular velocity or image displacement speed within the field of view, the effective temporal variation of its image features at the pixel scale can reach the hundred-hertz order of magnitude. In accordance with the Nyquist sampling criterion, to avoid temporal aliasing and accurately reconstruct the dynamic characteristics of the target, the effective frame rate of the projected scene must be at least twice the scene bandwidth, i.e., 200 Hz or higher. Meanwhile, considering the detector integration time and system timing margin, a higher frame rate is often necessary to mitigate dynamic distortion. This directly drives the demand for high-resolution, high-frame-rate scene projection capabilities, so as to eliminate motion blur and temporal aliasing and achieve high-fidelity hardware-in-the-loop testing. In addition, current infrared scene simulation systems are mostly designed for a single waveband. However, in practical detection environments, targets often emit visible light, short-wave infrared (SWIR), and mid-wave infrared (MWIR) signals simultaneously, rendering single-waveband simulation inadequate for meeting the performance calibration requirements of multi-waveband detectors [6,7]. Therefore, the development of a multi-waveband infrared scene simulation and testing system is crucial for realistically replicating the actual operating scenarios of detection systems in laboratory environments and ensuring the validity of tests [8,9].

The Digital Micromirror Device (DMD) has emerged as the core component of various scene simulators and been widely adopted, thanks to its prominent advantages including high frame rate, high contrast ratio, high brightness, and rapid response [10,11,12]. Nevertheless, the active area size of DMD is constrained by the number of micromirrors and micromirror pitch, which is essentially a technical limitation of existing manufacturing processes and has become the key factor restricting the further enhancement of system performance [13]. In existing research, the mainstream strategy for expanding the DMD active area is to improve display resolution: for instance, Zhang et al. [14] achieved doubled resolution via a time-division multiplexing scheme, but the frame rate was reduced to 60 Hz; TI’s DLP660TE [15] realizes 4K display by virtue of XPR technology, with the frame rate also limited to 60 Hz, which is far from satisfying the high frame rate requirement for highly dynamic scene simulation and thus leads to dynamic blur that seriously degrades the accuracy of target recognition. Meanwhile, even though existing commercial spatial light modulators (SLMs) possess a high pixel count, they are often constrained by frame rate, contrast ratio, spectral compatibility, and other factors, making it challenging to simultaneously meet the comprehensive requirements of large active area, high frame rate, and multi-waveband synchronous projection. If tiled display is employed to achieve a large active area, the issue of time synchronization among multiple channels arises, which tends to induce uneven brightness and target misalignment in the stitching region, severely impairing the validity of simulation. Consequently, when addressing the detector matching requirements of 1920×1080 resolution and above, coupled with high frame rate demands, system design often has to make trade-offs between key performance metrics such as active area size and output frame rate, making it difficult to optimize all indicators simultaneously.

To tackle the aforementioned problems, this paper proposes a large-target-surface, high-frame-rate display method based on the synergistic operation of multiple DMDs, which is suitable for hardware-in-the-loop optical simulation systems. This method configures DMD groups by waveband (four DMDs each for visible light, SWIR, and MWIR) and integrates them with a beam splitting and combining optical system, enabling synchronous scene simulation covering these three wavebands. Meanwhile, it adopts the technical route of multi-DMD stitching and synchronous display, eliminating the need to improve micromirror integration density through chip manufacturing. This thereby avoids the process complexity and cost escalation associated with resolution enhancement, and significantly expands the active area size without sacrificing resolution and frame rate, ultimately realizing a large-target-surface, high-frame-rate, multi-waveband infrared scene simulation system. Experimental verification demonstrates that the system can synchronously display 8-bit grayscale images at a frame rate of 400 Hz, with an active area size four times that of a single DMD, and the synchronization error between individual DMDs is less than 10 ns. This system can meet the multi-waveband performance calibration requirements of satellite remote sensing, airborne electro-optical reconnaissance, and related equipment, while enabling high-fidelity simulation of the dynamic infrared characteristics of high-speed targets in a standardized and reproducible laboratory environment.

## 2. Principle

**Figure 1:**
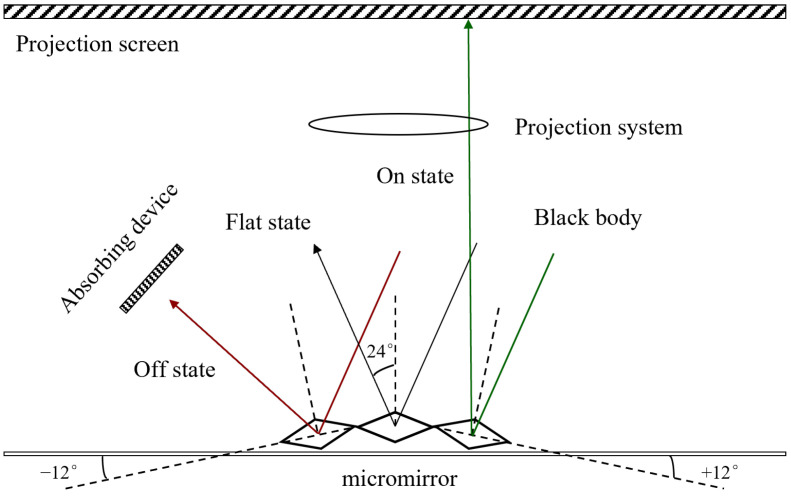
Schematic diagram of DMD operation.

A DMD consists of numerous small aluminum-made reflective mirrors. Each mirror is regarded as a pixel and can deflect ±12° around its diagonal [16]. These deflections respectively correspond to three states, the on state (+12°), the floating state (0°), and the off state (−12°), as shown in Figure 1. The on state and the off state of the micromirror can also be considered as the “bright” and “dark” states respectively, corresponding to the binary “1” and “0” of the control signal.

### 2.1. DMD Grayscale Modulation

Since the working principle of DMD is based on the “on” and “off” states of micromirrors, it can only display binary images at the same time. To enable DMD to display images with a high grayscale level, grayscale modulation of DMD is required. Common grayscale modulation methods include the spatial grayscale modulation method, the frame grayscale modulation method, and the PWM method [17]. The spatial grayscale modulation method achieves grayscale modulation by sacrificing spatial resolution. That is, increasing the grayscale level will reduce the resolution of the displayed image. The frame grayscale modulation method utilizes the superposition effect of multiple sub-frames to achieve grayscale modulation. However, increasing the number of sub-frames will limit the display frame rate. In contrast, the PWM method can increase the display frame rate without reducing the resolution, so it is more suitable for realizing the display of high-grayscale images.

Similar to the frame grayscale modulation method, the PWM method also performs a superposition operation on multiple sub-frames. However, this method is achieved by changing the ratio of the time a pixel is in the “on” state to the integration time (i.e., the duty cycle). In this way, the superposition of sub-frames does not limit the frame rate. Take an 8-bit grayscale image as an example (with a total of 256 grayscale levels). At this time, the duty cycle of PWM is closely related to the grayscale level. Suppose the time of one image frame is taken as the period *T* of PWM and is divided into 256 time slices. For a certain pixel, if it is to display the *n*th grayscale level (*n* ranges from 0 to 255), $t_{on}$ is set as Equation (1):

(1) $$t_{on}=\frac{n}{255}T$$

The duty cycle *D* is set as Equation (2).

(2) $$D=\frac{n}{255}\times 100\%$$

In this way, by changing the PWM duty cycle of the micromirror corresponding to each pixel, the time proportion of its reflected light can be precisely controlled. As a result, different grayscale effects can be presented, and ultimately, high-quality image display can be achieved.

To simplify the flipping operation of the DMD, the display image is segmented bit by bit here. The display time of each sub-frame depends on the bit number of that sub-frame. Taking the display of an 8-bit grayscale image as an example, the image is first segmented into eight binary images according to bits. Suppose the display time of the 0-bit-plane image is $t_{base}$; then the display time of the n-bit-plane image is $2^{n}\cdot t_{base}$. Displaying an 8-bit grayscale image using the PWM method based on the bit-plane slicing technique only requires the micromirrors to flip eight times. This greatly reduces the flipping time of the DMD and increases the display frame rate of the image.

### 2.2. Synchronized Display

Synchronized display serves as the core foundation of the system. Its essence lies in achieving strict temporal alignment of micromirror deflection actions across multiple DMDs through signal timing consistency control and device response calibration. Only when all target pixels on every DMD enter the “on” or “off” state simultaneously can abnormal light intensity overlay caused by misaligned micromirrors in the stitching region be avoided, ensuring the integrity and consistency of the projected image. Particularly in high-frame-rate dynamic scene simulations, synchronization precision should be sufficiently smaller than the bit-plane switching. Failure to do so causes positional shifts in moving targets, reducing the fidelity of hardware-in-the-loop simulations in replicating real-world environments.

**Figure 2:**
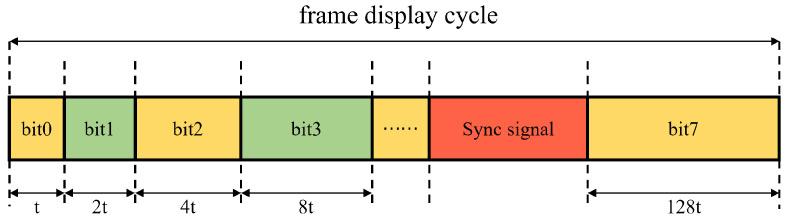
Schematic diagram of PWM.

To achieve the synchronous display function of the system, this paper designs a multi-level synchronous control mechanism based on synchronous pulse signals. The system operation mechanism is based on the PWM method shown in Figure 2. The time interval from the start of the 0-bit plane of the first image frame to the start of the 0-bit plane of the next image frame is defined as the frame cycle of the DMD. The system generates a synchronous pulse signal once within each frame display cycle to achieve precise synchronization. Specifically, during the display process of the 8-bit planes of each image frame, the first seven bit planes operate sequentially according to Figure 2, and the 8th bit-plane uses a synchronous pulse trigger mechanism for display. That is, the display of the 8th bit-plane is triggered only after receiving the synchronous pulse signal, thus ensuring the synchronization of the system.

#### 2.2.1. Basis for the Design of Synchronous Pulse Signal

The timing of the synchronous pulse signal trigger is crucial to ensuring synchronization accuracy. This study places it between bit6 and bit7 rather than between other bit planes. This design choice represents the optimal solution based on a comprehensive consideration of the following three aspects: the temporal characteristics of the PWM bit planes, the impact of synchronization operations on final image quality, and the overall system robustness. The detailed explanation is as follows:

1. Maximizing synchronization timing margin to ensure operational stability: In 8-bit PWM driving, the display time of each bit plane increases exponentially. The most significant bit (bit7) has the longest duration, accounting for approximately 50% (128/255) of the total frame period. Placing the synchronization point between bit6 and bit7 means the system aligns all DMDs before entering this longest and most critical display segment. This approach provides the most ample and stable time window for receiving, processing, and responding to the synchronization signal. It effectively absorbs minor delay variations caused by signal transmission and clock jitter across multiple boards, thereby significantly reducing the risk of synchronization mis-triggering or timing conflicts. In contrast, placing the synchronization point within the early bit planes (e.g., bit0–bit2), where display times are extremely short and interspersed with operations like “block clear,” leaves almost no redundancy to accommodate such delays, making synchronization failure or grayscale information errors highly likely.
2. Minimizing the impact of synchronization error on final image quality: Our system targets integrating photodetectors, whose output grayscale value is directly related to the integral of received optical flux during the exposure time. Bit7, as the highest-weight bit, contributes the dominant portion of the optical energy. Consequently, synchronization errors occurring during the bit7 period have the most significant impact on the final image’s grayscale uniformity. By precisely anchoring the synchronization point at the start instant of bit7, we ensure all DMDs synchronously begin this most energy-significant display process. This fundamentally prevents optical energy misalignment caused by unsynchronized start times of the bit7 segment across different DMDs. Comparatively, placing the sync point between bit3 and bit6, while offering a better timing margin, cannot guarantee strict phase alignment for the bit7 segment. Placing it between frames (i.e., after bit7) only achieves frame-level synchronization and cannot constrain the start time of the bit7 segment within a single frame, potentially still leading to visible brightness inconsistency in stitched regions.

In summary, the design of placing the synchronization pulse between bit6 and bit7 is superior to other candidate schemes in terms of both operational reliability and imaging quality assurance. It fully leverages the temporal structure of PWM, maximizes system robustness, and precisely controls synchronization accuracy for the most temporally sensitive segment. This effectively meets the stringent timing consistency requirements of high-frame-rate, large-target-surface, multi-band scene simulation systems. It should be noted that the aforementioned design principle of “anchoring the synchronous pulse at the starting boundary of the MSB” is not specific to the hardware platform described in this paper, but has universal significance for DMD drivers that implement PWM grayscale synthesis using bit-plane sequences. The fundamental basis for this is as follows: In a typical binary weighted bit-plane structure, the MSB segment contributes the most to the energy integration within a frame, so the inconsistency in the starting phase of the MSB is most likely to manifest as visible brightness differences or dynamic target misalignment in the stitching area. Anchoring the synchronous constraint at the starting boundary of the MSB can achieve the strongest constraint for the most sensitive period with the minimum cost of synchronous control, thereby improving the consistency and robustness of the stitching display.

#### 2.2.2. Composition of Synchronization Error and Optimization Strategies

Assuming that these contributions are mutually independent, the total synchronization error can be approximated by the root-sum-square (RSS) method. Based on this mechanism, the total synchronization error $\Delta t_{total}$ can be decomposed as shown in Equation (3):

(3) $$\Delta t_{total}=\sqrt{\Delta t_{trans}^{2}+\Delta t_{mirror}^{2}+\Delta t_{clk}^{2}}$$

Among them, $\Delta t_{trans}$ denotes the signal transmission delay error, $\Delta t_{mirror}$ the micromirror response error, and $\Delta t_{clk}$ the clock drift error. The physical causes of each error term are as follows:

1. $\Delta t_{trans}$ arises from the velocity characteristic of synchronous pulses propagating as electromagnetic waves in FPGA internal wiring and SMA cables (approximately $2\times 10^{8}$ m/s). Differences in wiring and cable lengths lead to transmission delays.
2. $\Delta t_{mirror}$ is determined by the inherent mechanical deflection characteristics of DMD micromirrors. The physical process of a DLP9500 micromirror flipping from the “off” state to the “on” state takes about 3 μs [18]. Affected by MEMS manufacturing tolerances, individual micromirrors exhibit minor differences in mechanical performance. However, since the refresh of DMD bit planes is triggered by a globally synchronized timing sequence, the relative deviation of flipping moments among micromirrors in a single event is far smaller than the flipping duration. Thus, this effect will be neglected in the system-level timing model of this paper.
3. $\Delta t_{clk}$ originates from the thermal noise characteristics of the crystal oscillators in the FPGA and DMD driver boards—for every 1 °C change in temperature, the crystal oscillator frequency shifts by approximately 10 ppm, resulting in cumulative deviations in the clock cycle with temperature.

Thus, the logic design of the main control board adopts an architecture that binds the synchronous pulse signal to the internal registers of the FPGA pin resource IOB (Input/Output Block) [19,20]. This design ensures that all output signals are processed by flip-flops when leaving the FPGA, thus guaranteeing the complete consistency of all synchronized signals in the time dimension and effectively avoiding the timing deviation caused by the internal wiring delay of the FPGA. Meanwhile, each group of DMDs uses SMA cables of consistent length to transmit synchronous pulse signals, reducing the signal transmission delay error.

To further optimize the system synchronization accuracy, this paper designs a multi-level synchronized control mechanism based on the internal self-calibration of the DMD driver module and the centralized time calibration of the main control computer. This mechanism collaboratively optimizes from the hardware and the system level, effectively improving the time synchronization performance of the system. The specific implementation scheme is as follows:

At the hardware level, the DMD driver module is integrated with a high-precision self-calibration system, which can control the time error within one part per million (1 ppm); that is, the synchronization error within a time scale of 1 second is within ±1 μs. This high-precision self-calibration ability mainly relies on the precise clock management unit and signal processing circuit inside the module. By accurately adjusting the system clock signal and strictly controlling the data transmission timing, the system realizes dynamic self-adjustment during operation, significantly reducing internal circuit delay and clock drift, and providing reliable hardware support for the system’s synchronization performance.

From the system level, this paper designs a centralized time-calibration mechanism based on bus communication. This mechanism initiates a global time-calibration operation in a minute-based cycle, giving full play to the high efficiency and stability of bus communication. The main control computer quickly transmits the precise time reference to each DMD driver module through this mechanism. After receiving the time-calibration signal, each module immediately calibrates its internal clock in real time according to the time base provided by the main control computer. This hierarchical time synchronization architecture effectively ensures precise temporal synchronization of all DMD driver modules, reduces clock drift errors, and truly meets the system’s strict technical requirements for synchronization error.

By combining the high-precision self-calibration at the hardware level and the centralized time calibration at the system level as described above, the multi-level control mechanism proposed in this paper significantly improves the synchronization performance of the system, providing a reliable technical guarantee for achieving high-precision synchronized display and ensuring the stability of system operation and the reliability of function realization. By establishing a mapping relationship of “error term → constraint means → verification index”, the synchronization scheme is organized into a set of reproducible and engineering-able evaluation processes, rather than empirical sequential debugging. For different platforms, this process remains unchanged, and mainly requires re-calibration and verification based on the device and link parameters.

## 3. Materials and Methods

### 3.1. System Components

**Figure 3:**
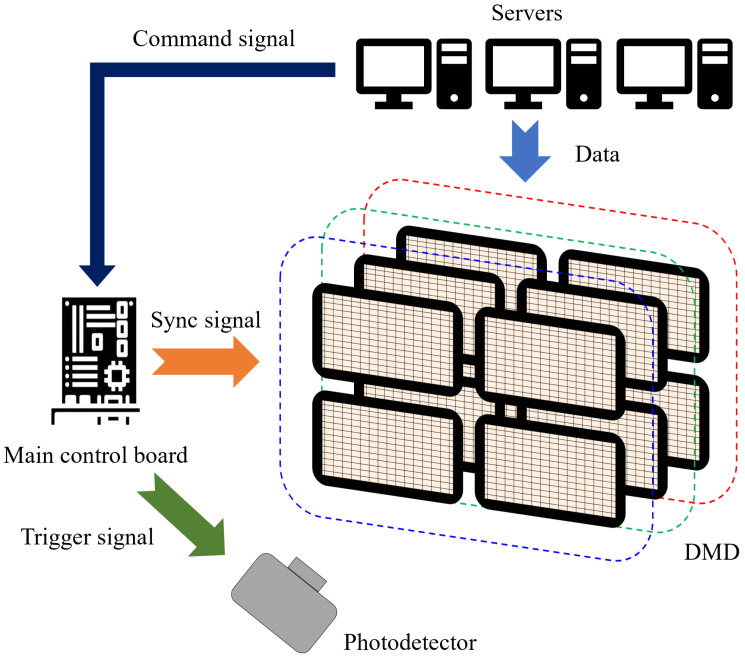
Structure of the DMD drive system.

The entire system is composed of three system servers, a main control board, twelve DMD driver boards, and twelve DLP9500 units. Communication between the servers and the DMD driver boards is carried out via a 10-Gigabit Ethernet, with optical fiber serving as the physical medium. The main control board externally connects 12 DMD driver boards through SMA cables. The DMD driver boards are connected to the DLP9500 via flexible PCBs. The system architecture is shown in Figure 3. Twelve DMDs are divided into 3 groups with 4 DMDs in each group for tiled display. The three tiled groups of DMDs respectively correspond to the visible light band, the SWIR band, and the MWIR band. Each server corresponds to one group of DMDs and is mainly used for storing image data and transmitting image data through the 10-Gigabit Ethernet. One of the servers, in addition to the above functions, also needs to communicate with the main control board via RS422 to send remote control commands. The function of the main control board is to parse the commands from the servers and send synchronous pulses and various remote control commands according to the server commands to complete the synchronization and control operations of the 12 DMD driver boards. Finally, 8-bit grayscale images are projected onto the photodetector on the target surface for imaging. When the integration time of the photodetector is asynchronous with the display time slot of the DMD, the images captured by the photodetector will suffer from aliasing, resulting in distorted image information. To address this technical challenge, the main control board designed in this study can generate precise trigger control signals and apply them to the photodetector. Through this triggering mechanism, strict timing synchronization between the integration period of the photodetector and the display time window of the DMD is achieved, thereby eliminating the interference of aliasing in image information.

### 3.2. Frame Rate Analysis

The system adopts the DLP9500 DMD chip from Texas Instruments, Dallas, TX, USA [18], whose relevant parameters are shown in Table 1:

Frame rate refers to the number of complete cycles of “data loading–micromirror flipping–stable display” completed by the DMD per unit time, and its upper limit is jointly determined by the mechanical response speed of the DMD and the circuit data transmission speed. The cycle of a DMD displaying one frame of the image using the PWM method consists of three parts: data loading time $t_{load}$, global reset time $t_{reset}$, and display hold time $t_{display}$, where $t_{display}=2^{n}t_{base}$, and n(0-7) represents the bit-plane number.

Since the DMD only supports row-addressing for data access and does not support column-addressing, the DMD memory is loaded by rows. Even if only one pixel in a row needs to be changed, the entire row must be loaded. For the DLP9500 1080p DMD chip, the micromirror array size is 1920×1080. It is divided into 15 blocks, with each block containing 72 rows [18]. Each row actually has 2048 memory cells, but no micromirrors are installed on the first 64 and the last 64 memory cells of each row. So, only 1920 micromirrors can be used in each row. However, when loading data, it needs to be done in units of 2048 bits. Therefore, when writing to a specified row address, 2048-bit data needs to be written at once. The DLP9500 uses two 32-bit-wide buses to complete row loading, and the data is loaded on the rising and falling edges of the data clock, that is, transmitted at Double Data Rate (DDR). Loading 2048-bit data for one row requires 32 clock edges, which is 16 clock cycles. Calculated based on the highest-rate 400 MHz clock supported by the DLP9500 model, the time required to load all 1080 rows of data is set as Equation (4):

(4) $$t_{load}=\frac{16}{400\;M}\times 72\times 15=43.2\times 10^{-6}\;s=43.2\;\mu s$$

For the DLP9500 1080p DMD chip, the global reset time $t_{reset}=4.5\;\mu s$ [18]. Since the micromirrors of the DMD chip require at least 8 μs to stabilize after flipping, during this period, the DMD chip cannot perform flipping operations but can carry out operations such as loading and displaying image information. Therefore, $t_{base}=8\;\mu s$ is selected here.

Since the display hold time of the first three bit planes is shorter than $t_{load}$, failure to force shutdown will result in the micromirrors remaining in the previous state when data of the next bit plane is loaded, causing grayscale superposition errors. Thus, a “block clear” operation needs to be added when the first three bit planes are displayed. This enables the first three bit planes to stop displaying after the display hold time and switch to the “off” state. For the DLP9500, the time required to execute a “block clear” command is the same as that of a row loading operation, which is 16 clock cycles. Two No-Operation (No-Ops) row cycles need to be run after the block clear command to complete the clearing of one block [21]. Therefore, the time required for the “block clear” operation is set as Equation (5):

(5) $$t_{blockclear}=\frac{16+32}{400\;M}\times 15=1.8\times 10^{-6}\;s=1.8\;\mu s$$

It can be observed here that the “block clear” command is 24 times faster than the row loading operation and can be included within $t_{base}$. Except for the first three bit planes that require “block clear” and “reset” operations, the data loading times of the remaining bit planes are all included within the display hold time of the previous bit plane. Thus, the data loading of the next bit plane can be completed during the display hold phase.

**Figure 4:**
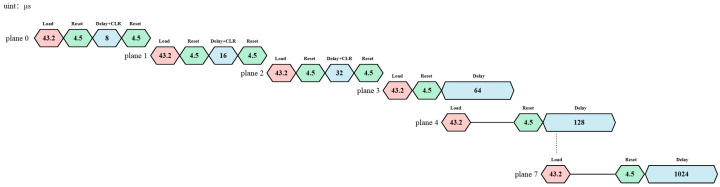
Timing diagram of DMD based on PWM and “block clearing” method.

According to the timing analysis in Figure 4, the time required to display one frame of an 8-bit grayscale image based on the PWM method can be calculated as follows: The display time of the bit planes is 255 times the time base $t_{base}$. The data loading times of the first three bit planes are not included in the display hold time. Therefore, the frame cycle contains four data loading times. From a cyclic perspective, the loading of a 0-bit plane can be included in the display of 7-bit plane. Thus, when calculating the frame cycle, only three data loading times are counted. Meanwhile, the display processes for the first three bit planes each include 2 reset waiting times, and the display processes for the remaining bit planes each include 1 reset waiting time. Therefore, the frame cycle contains 11 reset waiting times. The display cycle based on the PWM method is set as Equation (6):

(6) $$T=3t_{load}+\left(2^{8}-1\right)t_{base}+11t_{reset}=3\times 43.2+255\times 8+11\times 4.5=2219.1\;\mu s$$

The display frame rate is set as Equation (7):

(7) $$F=\frac{1}{T}=450.6\;\mathrm{Hz}$$

In conclusion, the highest display frame rate of an 8-bit grayscale image based on the PWM method can reach up to 450.6 Hz.

The above frame rate analysis leverages a common hidden-loading idea, i.e., scheduling the data loading of the next bit plane/sub-frame into display window to reduce explicit loading overhead. This strategy is not unconditionally applicable to all SLM platforms, because its feasibility depends on the controller timing model and the on-chip memory organization. In general, it requires that (i) display holding and memory writing can overlap (the controller allows writing the next sub-frame to on-chip memory while the current bit plane is being held or during reset/settling, without disturbing the current optical state) and (ii) the available display window covers the required loading time, i.e., $t_{load}\leq t_{display}$. Otherwise, additional explicit loading time must be inserted and the achievable frame rate must be re-evaluated.

In this paper, we instantiate the timing parameters using the DLP9500 + DLPC410 platform; thus, numerical values such as $t_{load}$, $t_{reset}$, $t_{display}$ and the bit-plane organization are platform-dependent. Nevertheless, the scheduling principle and feasibility criterion are transferable: for a different DMD and controller combination, one can substitute the platform-specific timing constants and bit-plane structure into the same analysis to verify whether the desired frame rate is achievable.

### 3.3. Experimental System Construction

**Figure 5:**
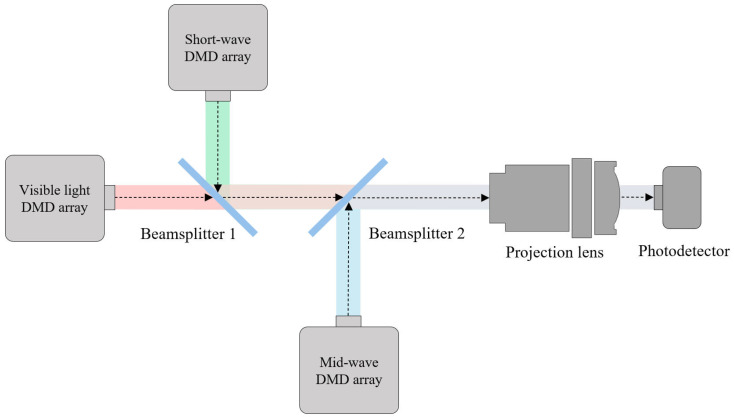
Schematic diagram of the optical path of the experimental system, where red, green and blue represent light beams in the visible, short-wave and mid-wave bands, respectively. Colored regions between Beamsplitter 1/2 and between Beamsplitter 2 and the Projection lens indicate the combination of these wave bands. All colors denote wave bands only and do not reflect their actual chromaticity.

The schematic diagram of the optical path of the experimental system is shown in Figure 5. Beamsplitter 1, beamsplitter 2, the projection lens, and the photodetector are sequentially fixed on the main optical path. The DMD group for the visible light band and the DMD group for the short-wave band are respectively fixed on the left side and the upper position of beamsplitter 1. The DMD group for the mid-wave band is fixed at the lower position of beamsplitter 2. Beamsplitter 1 transmits light in the visible band and reflects light in the short-wave band. Beamsplitter 2 transmits light in the visible and short-wave bands while reflecting light in the mid-wave band. The light sources of each band enter the main optical path after being modulated by the DMD, are combined by the two beam splitters, and then projected onto the photodetector through the projection lens.

**Figure 6:**
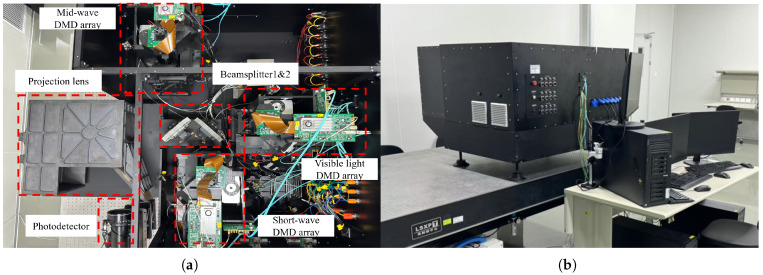
Physical diagram of the experimental system. (**a**) DMD groups of three bands and the combined light system. (**b**) The overall appearance of the system.

The experimental system adopts a large-target-surface and high-frame-rate display method with the collaboration of multiple DMDs. As shown in Figure 6a, the system includes DMD groups for three bands, light sources, beamsplitters, projection lenses, and other components. The photodetectors used in the experiment are a visible light band photodetector (resolution: 3840 × 2160, pixel size: 3.45 μm), a short-wave band photodetector (resolution: 2560 × 2048, pixel size: 3.45 μm), and a mid-wave band photodetector (resolution: 640 × 512, pixel size: 15 μm). The photodetector is mounted on a high-precision manual platform displacement stage and a manual lifting stage. These can be used in combination to enable movement in a three-dimensional space, which makes it more convenient for the detector to receive images.

## 4. Results

### 4.1. Projection Test

#### 4.1.1. Illumination Uniformity Test

Here, the Uniformity Index (UI) is used to evaluate the uniformity of the illuminance distribution [22]. UI is an important indicator for measuring the consistency of the luminance distribution in the projection area, and its calculation formula is set as Equation (8):

(8) $$UI=\frac{E_{min}}{E_{mean}}\times 100\%$$

where $E_{min}$ represents the minimum illuminance value within the measurement area, and $E_{mean}$ represents the mean illuminance value within the measurement area. The result calculated by this formula can intuitively reflect the uniformity of the illuminance within the illuminated area. The closer the value is to 100%, the more uniform the illuminance distribution; conversely, the smaller the value, the greater the illuminance difference.

**Figure 7:**
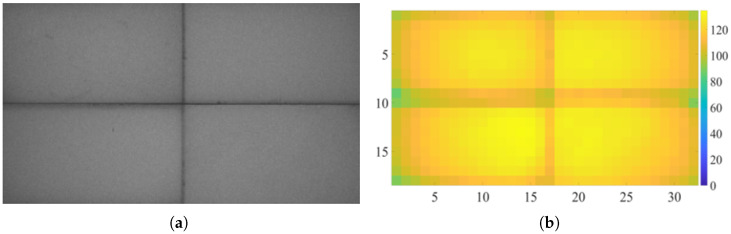
Illuminance test diagrams of the projection area. (**a**) The brightness distribution of the projection area. (**b**) A two-dimensional heat map of the brightness distribution.

A solid-color image with a grayscale value of 128 was used as the system input for the illuminance uniformity test. The output image of the photodetector is shown in Figure 7a. It can be seen that the system can achieve projection display with high illuminance uniformity.

To evenly present the luminance distribution within the projection area, the output image was uniformly divided into 32 (horizontal direction) × 18 (vertical direction) rectangular sub-blocks, and the average grayscale value of each sub-block was calculated. By traversing all sub-blocks, the minimum illuminance value and the average illuminance value were determined, and thus the illuminance UI was calculated. Considering that the black seams generated by tiling would have a significant impact on the illuminance uniformity test, the sub-blocks where the black seams were located were ignored during the calculation. Finally, the results were visualized in the form of a two-dimensional heatmap, as shown in Figure 7b. The UI was more intuitively presented through the heatmap, enabling the evaluation of the uniformity of the illuminance distribution.

The key factor affecting illuminance uniformity lies in the imaging light beam. Light beams from different positions of the DMD are restricted by the aperture during propagation, and the chief rays in the edge regions are more prone to occlusion. This leads to a reduction in the effective light-transmitting aperture and a lower transmitted luminous flux compared to the central region, ultimately forming an illuminance field characterized by “brighter center and darker edges”—a distribution consistent with the features observed in two-dimensional heat maps. On the other hand, if the light source itself exhibits spatial or angular inhomogeneity in luminous intensity, it will further exacerbate the non-uniformity of illuminance. To further improve uniformity, a light source with more stable luminous distribution should be selected, and a light homogenizer should be used in conjunction to mitigate the impact of intensity fluctuations.

The UI of the projection system was calculated to be 84.1%. It can be concluded that the projection system has high illuminance uniformity, can achieve a stable illuminance distribution in the overall area, and is conducive to the system achieving good reliability and repeatability.

#### 4.1.2. Grayscale Image Display Test

**Figure 8:**
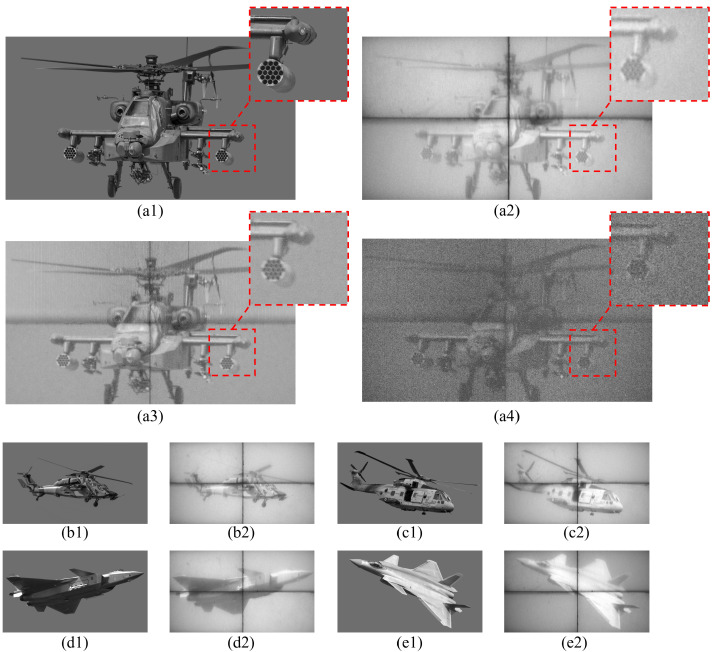
Comparison of original grayscale images [23,24,25,26,27] with photodetector imaging: (**a1**) original grayscale image; (**a2**) visible light band photodetector imaging; (**a3**) short-wave band photodetector imaging; (**a4**) mid-wave band photodetector imaging; (**b1**) original grayscale image; (**b2**) visible light band photodetector imaging; (**c1**) original grayscale image; (**c2**) visible light band photodetector imaging; (**d1**) original grayscale image; (**d2**) visible light band photodetector imaging; (**e1**) original grayscale image; (**e2**) visible light band photodetector imaging.

The system was tested for the display of static images, and the experimental results are shown in Figure 8 below. The results indicate that the system can correctly display grayscale images. The details of the original image are accurately and completely reflected, demonstrating the system’s ability to effectively reproduce complex details.

**Figure 9:**
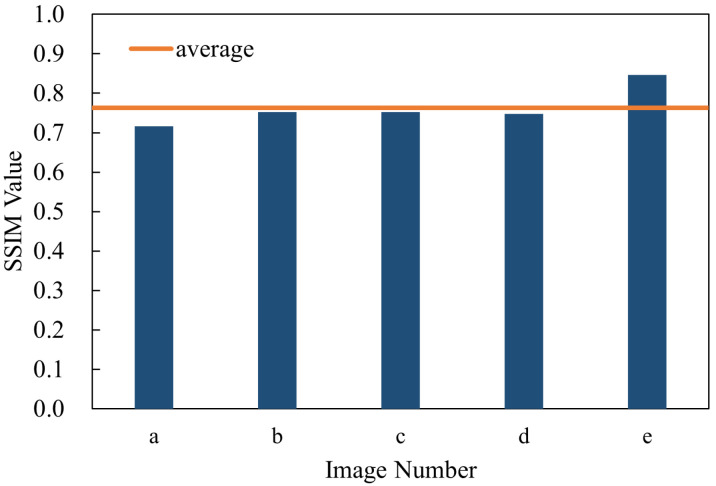
SSIM values of visible light band images calculated from the data of subfigures a to e in Figure 8.

The SSIM function in MATLAB R2022a (MathWorks, Natick, MA, USA) is utilized to assess the structural similarity between the images. The SSIM algorithm on which this function is based was proposed by Wang et al. [28]. It is based on the perception model of the Human Visual System (HVS) and can more accurately reflect the image quality. Compared with traditional pixel-level error measurement methods (such as Mean Squared Error, MSE, or Peak Signal-to-Noise Ratio, PSNR), SSIM is more in line with the human visual experience. SSIM measures the similarity between two images from three aspects: luminance, contrast, and structure. The closer its value is to 1, the more similar the two images are. The images captured by the visible light band photodetector are used here, and the SSIM values of multiple images calculated by calling the MATLAB function are shown in Figure 9. Based on the analysis of the calculated SSIM values, it can be concluded that the projection system has high imaging quality, can more accurately restore the structure and details of the original image, and the system has good repeatability and reliability.

### 4.2. Experimental Verification

#### 4.2.1. Frame Rate Test

The display frame rate can be tested by projecting a special static image [29]. The server transmits a uniform scene with a grayscale value of 128 to the DMD. After the image is segmented into bit planes, the pixel data of bit planes 0 to 6 are all “0”, and the pixel data of bit plane 7 are all “1”. Therefore, when displaying the test image, a light pulse is generated in the display of each frame, and the frequency of the light pulse is the DMD display frame rate based on the PWM method.

**Figure 10:**
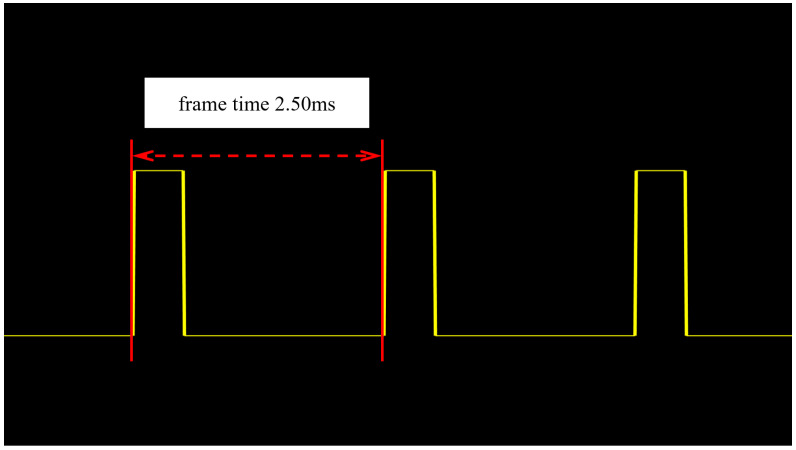
Voltage waveform of the silicon photocell.

A silicon photocell can convert the optical signal into a voltage signal. Therefore, the light pulse signal generated during the display can be detected using a silicon photocell. In the frame rate test experiment, the silicon photocell is connected in series with a load resistor. By simply detecting the voltage across the load resistor, the detection of the light pulse frequency can be achieved. The test results are shown in Figure 10, which indicates that the display frame rate of the simulation system is 400 Hz. The reason why the measured frame rate is lower than the calculated one in Section 3.2 is that the system introduces a synchronization signal in order to synchronize the display, and the frame rate of the synchronization signal is set to 400 Hz. Setting it at the relatively more conservative 400 Hz can effectively avoid potential timing errors that might occur if the frame rate were set at the more extreme value of 450.6 Hz, ensuring the stable operation of the system.

**Figure 11:**
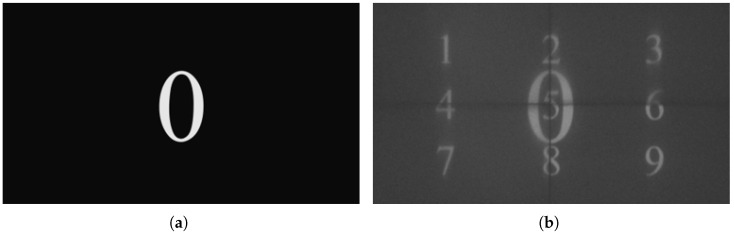
Images for verifying the frame rate. (**a**) Image of the digit “0”. (**b**) Projected images including digits from “0” to “9”.

Here, the frame rate obtained in Figure 10 is verified by projecting a test video composed of the digits from 0 to 9. As shown in Figure 11a, taking the image of the digit “1” as an example, ten-digit images at different positions are projected sequentially. The test image is projected onto the photodetector, and the integration time of the target detector also needs to be adjusted. By analyzing the waveform in Figure 10, the projection time of the ten images is approximately 25 ms. To account for system errors, the integration time of the photodetector should be slightly longer than the total projection time of the ten images. In the experiment, the integration time of the photodetector is set to 26 ms (slightly longer than 10 times 2.5 ms). In the external trigger mode, the DMD system and the photodetector are synchronously driven by an external trigger signal. If all the digit images from “0” to “9” can be clearly imaged, it proves that the projection of 10 images has been successfully completed within the integration time, and the frame rate has reached the expected target.

The projection result was captured by the photodetector, and the obtained photo is shown in Figure 11b. As can be seen from the photo, the projection frame rate of the system reaches at least 384 Hz ($\frac{10^{3}\;\times \;10}{26}$), which basically meets the expected frame rate requirements.

#### 4.2.2. Synchronization Error Test

To verify the synchronized display performance of the system, we designed a test method based on synchronous pulse counting and a millisecond-level counter. The count of received synchronous pulses is set in the logic, and a millisecond-level counter is also set up. This counter is cleared when a synchronous pulse is received, meaning that it only records the interval between two synchronous pulses.

When sending a reset instruction for each bit plane, the count of the current synchronous pulse and the count of the millisecond-level counter are registered. That is, the display time of each bit plane can be expressed as the number of the synchronous pulse and the offset in milliseconds. The time required for resetting the first four bit planes of each frame is recorded using debugging tools. One DMD board of the first server and one board selected from each of the other two servers are chosen for the test. The test results of the first board of the first server are the same as those of the first board of the second server. Similarly, three frames are respectively selected for testing between every two boards, and all the test results are identical. This result indicates that the DMD boards in the system can achieve precise synchronization at the frame level, laying the foundation for the subsequent multi-DMD collaborative display.

**Figure 12:**
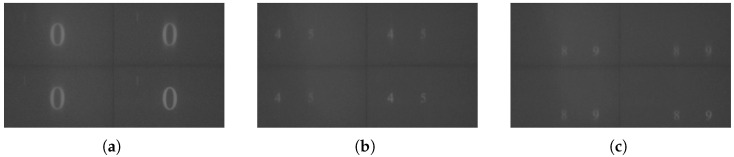
Projected video captures at different moments: (**a**) Moment 1; (**b**) Moment 2; (**c**) Moment 3.

To verify the synchronization error between the DMDs, all four DMDs projected the test video in Section 4.2.1. The projected images were captured by a camera, and the resulting photograph of the projected images is shown in Figure 12. It can be observed that the images displayed by the four DMDs exhibit high consistency, indicating that synchronous display can be achieved among the DMDs in the system.

**Figure 13:**
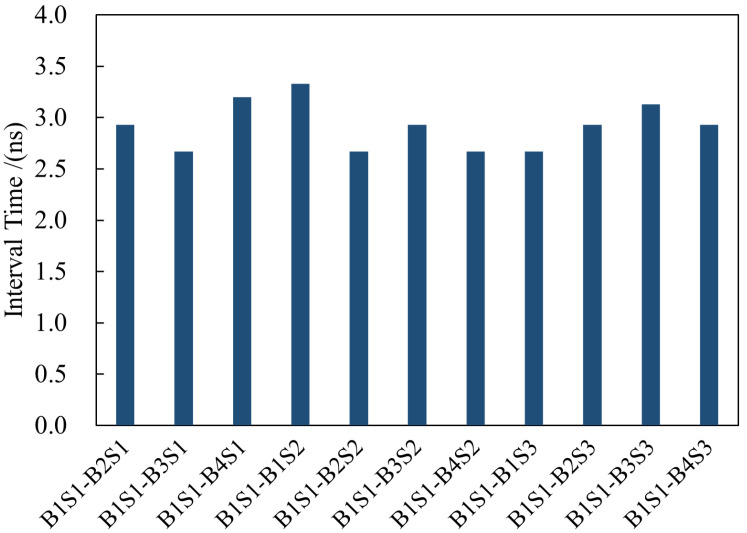
Synchronization error test results.

To further verify the long-term stability and synchronization robustness of the system during prolonged operation, measurements were taken at the receiving end of the SMA cables on each board using an oscilloscope after ten hours of operation. The results are shown in Figure 13.

Therefore, it can be concluded that the synchronization error between the various DMDs in the system is less than 10 ns, and the system has long-term stability.

## 5. Discussion

This study has successfully developed and validated a large-target-surface, high-frame-rate display technology based on the synergistic operation of DMDs. It significantly expands the effective target surface size without sacrificing frame rate, demonstrating substantial application potential in the field of hardware-in-the-loop optical simulation. The core innovation of this technology lies in the combination of PWM and multi-DMD synchronous modulation schemes, which synergistically optimize the system’s target surface size and frame rate performance. Compared with traditional single-DMD projection systems, this technology achieves simultaneous improvements in target surface size and high frame rate while maintaining a high grayscale level, making it of great value for high-precision simulation of high-speed dynamic scenes.

During the research process, we also identified several aspects of the system that require further optimization:

1. Black seam issue caused by DMD tiling: Due to the geometric constraints of the optomechanical structure and splicing prisms, overlapping fusion at the boundaries is not permitted. This results in physical gaps in the system due to manufacturing and mechanical assembly tolerances of the splicing prisms, forming black seams that cannot be covered by the reflected light of the micromirrors. Based on the visible light band imaging in Figure 8, the typical width of these black seams is approximately 15 pixels. In Fourier-domain processing scenarios (such as coherent optical imaging and spectral modulation), such structural features may introduce additional frequency components, leading to artifacts in the frequency-domain distribution and affecting the accuracy of subsequent signal processing. However, it is important to clarify that the core application scenario of this system is hardware-in-the-loop scene simulation projection, whose evaluation and use are usually conducted in the image-plane intensity domain. The key performance indicators focus on large target surface, high frame rate, multi-band compatibility, and synchronous display. Fourier-domain optical processing is generally not required in this scenario, so the impact of the black seams on the core application scenario is controllable and can be further mitigated through engineering measures, and it is not a performance bottleneck for the current core application scenario.
2. Trade-off between frame rate improvement and image brightness: As the frame rate increases, the brightness of the projected image decreases correspondingly. This is mainly due to the compression of the display time of each sub-frame in PWM modulation, resulting in a shorter effective light reflection duration of the pixels. In the future, the light utilization efficiency can be improved by increasing the light source power and optimizing the light-gathering efficiency of the optical system to alleviate the brightness attenuation issue.
3. Relatively high imaging noise in the MWIR band: Images captured in the MWIR band exhibit relatively obvious noise, primarily due to the low output energy of the system in this band. Subsequently, the signal intensity of this band can be enhanced and noise interference reduced by optimizing the selection of MWIR light sources and improving optical filtering schemes.

To address the aforementioned issues, future research will focus on optimizing the following aspects:

1. In-depth research on black seam suppression technology: A feasible approach involves adopting a scheme that combines fixed seam masks (i.e., invalid-region maps) with gradual transition—where fixed seam masks are obtained through one-time calibration and smooth window functions are applied to seam-adjacent areas to realize gradual brightness transitions from effective display regions to seam regions—and, for Fourier-domain processing scenarios, introducing an additional frequency-domain filtering step into the algorithm framework with optional strategies including boundary apodization, pre-compensation for known fixed textures and spatial filtering in the Fourier plane, with a trade-off between display resolution and light energy utilization efficiency being required for these measures; notably, these approaches do not alter the existing multi-DMD synchronization mechanism, and for specific applications demanding high-precision Fourier-domain processing, it is recommended to adopt the aforementioned suppression strategies or avoid aligning the seam direction with key frequency bands.
2. Optimization of light utilization efficiency and brightness: Improve the system’s light utilization efficiency by enhancing light source efficiency, upgrading manufacturing processes, or optimizing optical path design. On the basis of maintaining a large target surface and high grayscale level, further improve image brightness and contrast to meet a wider range of application requirements.
3. Balanced optimization of multi-band performance: Targeting the low energy issue in the MWIR band, optimize the band adaptability of the light source and optical system, reduce imaging noise in this band, and achieve balanced performance improvement across the three bands.

To clarify the technical positioning and advantages of the proposed system, we compared its key performance with that of mainstream SLMs, and the results are shown in Table 2.

As can be seen from the table, mainstream SLM solutions do offer options with 4K high resolution or high frame rates exceeding 1 kHz, but they usually struggle to simultaneously meet the comprehensive requirements of “high resolution + high frame rate + multi-band compatibility (especially MWIR)” under the same operating conditions. For instance, many 4K resolution solutions are often limited to a refresh rate of 60 Hz, while 1 kHz frame rate solutions typically sacrifice resolution or are mainly designed for the visible light band. The target scenario of the proposed system focuses on hardware-in-the-loop dynamic scene projection. It achieves a 4× target surface expansion at 8-bit grayscale and 400 Hz refresh rate, and realizes synchronous multi-band projection of visible light/SWIR/MWIR by leveraging the reflective characteristics of DMDs, thus filling the application gap of existing SLM products in multi-band, high-frame-rate, and large-target-surface projection scenarios.

In summary, the multi-DMD synergistic large-target-surface and high-frame-rate display method proposed in this study provides an effective technical solution for multi-band high-speed dynamic scene simulation in the aerospace field. Through targeted optimizations in subsequent research, its application scope can be further expanded to meet the requirements of more complex scenarios.

## 6. Conclusions

To meet the simulation requirements of a large area array, multiple bands, and a high frame rate, this paper proposes a high-frame-rate driving method for DMD based on the PWM method. Moreover, on the premise of a display frame rate of over 400 Hz, the synergistic operation of multiple DMDs is achieved. Combined with an optical system, DMD tiling and multi-band composite simulation can be realized. Experimental verification shows that the system is capable of synchronously displaying 8-bit grayscale images with a target surface size four times that of a single DMD at a frame rate of 400 Hz, and the synchronization error between each DMD is less than 10 ns.

## Figures and Tables

**Table 1:** DLP9500 related parameters.

Parameter Item	Parameter Content
Micromirror array size	1920×1080
Micromirror pitch (mm)	0.0108
Array diagonal (in)	0.95
Micromirror array orientation	Orthogonal
Display resolution (max)	1080p (1920×1080)
DCLK frequency(MHz)	400

**Table 2:** Comparison of Key Performances Between the Proposed System and Mainstream SLMs.

Comparison Item	Proposed Multi-DMD Synergistic System	Time-Division Multiplexing DMD [14]	XPR-Based DMD [15]	Hybrid Coding DMD [29]	GAEA-2.1 LCOS SLM [30]	Meadowlark 1024 × 1024 SLM [31]
Resolution/Pixel	3840 × 2160	3840 × 2160	3840 × 2160	1024 × 768	4160 × 2464	1024 × 1024
Frame Rate (8-bit grayscale)/Hz	400	60	60	2461	174	1400 (12-bit)
Modulation Capability	8-bit	8-bit	8-bit	8-bit	8-bit	12-bit
Spectral Compatibility	Visible, SWIR, MWIR (hybrid)	Visible	Visible	Visible	Visible-SWIR	Visible-SWIR

## Data Availability

The data included in this experiment are not yet publicly available but can be obtained from the author upon reasonable request.

## References

[1] Bhan R., Dhar V. (2019). Recent Infrared Detector Technologies, Applications, Trends and Development of HgCdTe Based Cooled Infrared Focal Plane Arrays and Their Characterization. Opto-Electron. Rev..

[2] Beuville E., Acton D., Corrales E., Drab J., Levy A., Merrill M., Peralta R., Ritchie W. (2007). High performance large infrared and visible astronomy arrays for low background applications: Instruments performance data and future developments at Raytheon. Proceedings of the Infrared Systems and Photoelectronic Technology II.

[3] Dhar N.K., Dat R., Sood A.K., Pyshkin S.L., Ballato J.M. (2013). Advances in Infrared Detector Array Technology. Optoelectronics.

[4] Bangs J., Langell M., Reddy M., Melkonian L., Johnson S., Elizondo L., Rybnicek K., Norton E., Jaworski F., Asbrock J., Andresen B.F., Fulop G.F., Norton P.R. (2011). Large format high-operability SWIR and MWIR focal plane array performance and capabilities. Proceedings of the Infrared Technology and Applications XXXVII.

[5] Yuan S., Shi L., Zhai Y., Yao B., Li F., Du Y. (2023). An Unsupervised Classification Method of Flight States for Hypersonic Targets Based on Hyperspectral Features. Chin. J. Aeronaut..

[6] Tang D., He Y., Huang F., Wang Q., Wang G. (2012). Study on Dynamic Infrared Scene Simulation Technique Based on Digital Micro-mirror Device. Infrared Technol..

[7] Meng L., Yang Y., Li H., Tang Y., Li Z., Qu Y., Zhao M., Li J. (2024). Design of a High-Frame-Rate and Large-Grayscale Simulation Projection System Based on Digital Micromirror Devices. Photonics.

[8] Hu W., Yuan X., He Y., Geng D., Tang D. (2014). Investigation of key technology about the multi-band dynamic infrared scene simulation system based on DMD. Opt. Instruments.

[9] Li Z., Gao Y., Zhang J. (2023). Multi-Spectral Complex Infrared Scene Projection Technology. Acta Opt. Sin..

[10] Goorden S.A., Bertolotti J., Mosk A.P. (2014). Superpixel-Based Spatial Amplitude and Phase Modulation Using a Digital Micromirror Device. Opt. Express.

[11] Xiong Z., Liu H., Chen R., Xu J., Li Q., Li J., Zhang W. (2018). Illumination Uniformity Improvement in Digital Micromirror Device Based Scanning Photolithography System. Opt. Express.

[12] Huang S., Ren B., Tang Y., Wu D., Pan J., Tian Z., Jiang C., Li Z., Huang J. (2024). Edge Smoothing Optimization Method in DMD Digital Lithography System Based on Dynamic Blur Matching Pixel Overlap Technique. Opt. Express.

[13] Zhuang Z., Ho H.P. (2020). Application of Digital Micromirror Devices (DMD) in Biomedical Instruments. J. Innov. Opt. Health Sci..

[14] Zhang Y., Surman P., He S. (2021). A Resolution-Enhanced Digital Micromirror Device (DMD) Projection System. IEEE Access.

[15] Incorporated T.I. DLP660TE 0.66 4K UHD Digital Micromirror Device. https://www.ti.com/lit/ds/symlink/dlp660te.pdf.

[16] Lee B. Introduction to ±12 Degree Orthogonal Digital Micromirror Devices (DMDs). https://www.ti.com/lit/an/dlpa008b/dlpa008b.pdf.

[17] Zhang K., Huang Y., Yan J., Sun L. (2013). Dynamic Infrared Scene Simulation Using Grayscale Modulation of Digital Micro-Mirror Device. Chin. J. Aeronaut..

[18] Incorporated T.I. DLP9500 DLP^®^ 0.95 1080p 2x LVDS Type A DMD. https://www.ti.com/lit/ds/symlink/dlp9500.pdf.

[19] Khairkar K., Khandekar P., Khambete Uday P., Jatana Design of FPGA Building Blocks Using LTspice®. Proceedings of the 2021 4th International Conference on Recent Developments in Control, Automation & Power Engineering (RDCAPE).

[20] Zhao Y., Wu L., Han X., Li Y., Zhang Q., Chen L., Zhang G., Li J., Yang B., Gao J. (2012). An IO Block Array in a Radiation-Hardened SOI SRAM-based FPGA. J. Semicond..

[21] Incorporated T.I. DLPC410 DMD Digital Controller. https://www.ti.com/lit/ds/symlink/dlpc410.pdf.

[22] Abboushi B., Irvin L., Rodriguez-Feo Bermudez E., Royer M. (2023). Evaluating Luminance Uniformity Metrics Using Online Experiments. LEUKOS.

[23] Commons W. File:RNLAF AH-64 Apache at the Oirschotse Heide Low Flying Area (36570605232).jpg—Wikimedia Commons, the Free Media Repository, 2024. https://commons.wikimedia.org/w/index.php?title=File:RNLAF_AH-64_Apache_at_the_Oirschotse_Heide_Low_Flying_Area_(36570605232).jpg&oldid=891401663.

[24] ho7dog. Helicopter, Airplane, Sky, Military, 2018. https://pixnio.com/transportation-vehicles/helicopters/helicopter-airplane-sky-military.

[25] akspic. Yellow and Red Helicopter Flying in The Sky Wallpaper, 2018. https://wallspic.com/image/3004-aerospace_engineering-aviation-helicopter_rotor-aircraft-air_force.

[26] Chengdu J-20, 2022. https://wallhaven.cc/w/6oxgm7.

[27] deskcar. J-20, 2017. http://www.deskcar.com/html/11411/13.shtml.

[28] Wang Z., Bovik A., Sheikh H., Simoncelli E. (2004). Image Quality Assessment: From Error Visibility to Structural Similarity. IEEE Trans. Image Process..

[29] Song R., Zhang T., Cui W. (2022). High Frame Rate Display Method Based on Composite Coding of Digital Micro Mirror and Illuminant. Semicond. Optoelectron..

[30] HOLOEYE Photonics AG Spatial-Light-Modulator-Brochure-HOLOEYE. https://holoeye.com/wp-content/uploads/Spatial-Light-Modulator-Brochure-HOLOEYE.pdf.

[31] Optics M. SLM-High-Speed-DS0325-HSP-UHSP-1024. https://www.meadowlark.com/wp-content/uploads/2025/04/SLM-High-Speed-DS0325-HSP-UHSP-1024.pdf.

